# Urine alkalization facilitates uric acid excretion

**DOI:** 10.1186/1475-2891-9-45

**Published:** 2010-10-19

**Authors:** Aya Kanbara, Masayuki Hakoda, Issei Seyama

**Affiliations:** 1 Department of Nutrition and Health Promotion, Faculty for Human Development, Hiroshima Jyogakuin University. 4-13-1 Ushita-higashi Higashi-ku Hiroshima 732-0063 Japan; 2 Department of Nutritional Sciences, Faculty of Human Ecology, Yasuda Women's University. 6-13-1 Yasuhigashi, Asaminami-ku, Hiroshima 731-0153 Japan

## Abstract

**Background:**

Increase in the incidence of hyperuricemia associated with gout as well as hypertension, renal diseases and cardiovascular diseases has been a public health concern. We examined the possibility of facilitated excretion of uric acid by change in urine pH by managing food materials.

**Methods:**

Within the framework of the Japanese government's health promotion program, we made recipes which consist of protein-rich and less vegetable-fruit food materials for H^+^-load (acid diet) and others composed of less protein but vegetable-fruit rich food materials (alkali diet). Healthy female students were enrolled in this consecutive 5-day study for each test. From whole-day collected urine, total volume, pH, organic acid, creatinine, uric acid and all cations (Na^+^,K^+^,Ca^2+^,Mg^2+^,NH_4_^+^) and anions (Cl^-^,SO_4_^2-^,PO_4_^-^) necessary for the estimation of acid-base balance were measured.

**Results:**

Urine pH reached a steady state 3 days after switching from ordinary daily diets to specified regimens. The amount of acid generated ([SO_4_^2-^] +organic acid-gut alkai) were linearly related with those of the excretion of acid (titratable acidity+ [NH_4_^+^] - [HCO_3_^-^]), indicating that H^+ ^in urine is generated by the metabolic degradation of food materials. Uric acid and excreted urine pH retained a linear relationship, where uric acid excretion increased from 302 mg/day at pH 5.9 to 413 mg/day at pH 6.5, despite the fact that the alkali diet contained a smaller purine load than the acid diet.

**Conclusion:**

We conclude that alkalization of urine by eating nutritionally well-designed food is effective for removing uric acid from the body.

## Background

Hyperuricemia is not only responsible for the generation of gout but also intimately associated with the incidence of cardiovascular diseases, renal diseases, hypertension and diabetes mellitus[1]. To improve hyperuricemia in the case of gout, almost all medical interventions taken at present rely on pharmacological tools, such as uricosuric medicines and xanthine oxidase inhibitors. Because pharmacological approaches are sometimes accompanied by side effects, alternative way to improve uric acid excretion would be helpful.

Hagos, Stein, Ugele, Burckhardt, and Bahn (2007)[2] have provided the general scheme of the commitment of human organic acid transporter 4 (hOAT4) in urate reabsorption in the proximal tubule. In their proposed mechanism similar to the hypothesis made by Guggino, Martin and Aronson (1983)[3] and Kahn and Weinman (1985)[4], hOAT4 can carry out inwardly directed urate transport in exchange with outwardly directed OH^- ^movement (urate/OH^- ^exchanger). The intracellular pH of the proximal tubule cells and, thereby, the OH^- ^gradient is maintained by the luminal sodium-proton exchanger (NHE3). The energy for Na^+ ^influx through NHE3 is provided by the favorable electrochemical gradient for Na^+ ^generated by the Na-K pump on the basolateral side. This characteristic of hOHT4 gave us a good hint to test whether one can provide an ideal tool without side effects to cure hyperuricemia. Since urine pH is determined by acid generation from food metabolism, we were able to generate urine having an intentional pH by eating designed diets. In this report, we will show that designed diets are effective to remove uric acid from the body, in such a way that alkaline urine is more favorable for removal than acid urine.

## Methods

### Subjects

Twenty-six healthy university female students ( 21-22 years old, 45-60 kg in body weight and 157-170 cm in height), who had no health problem records in the physical check up conducted by the university, participated in this study. The ethics committee at Hiroshima Jogakuin University approved the study protocol. All subjects signed informed consent documents. All participants lodged in the dormitory in the campus while the project was going. The condition of health of all participants was monitored by measuring body weight, the change of which was very limited during the projects (within less than 1% referred to body weight at the beginning of projects).

### Diet

Values for protein, energy and purine contents were extracted from the available data in the 5^th ^ed nutritional table issued by the Japanese Health, Welfare and Labor Department for all diets ingested by 26 subjects. Resultant calculation on the contents of amino acids in the diet which can generate an acid in the catabolic process, such as arginine, lysine, 1/2histidine, methionine and cystein, yielded 8039 mg/day in the alkali diet and 19458 mg/day in the acid diet. On the other hand, purine contents for diets were calculated to be 306 mg/day in the alkali diet and 533 mg/day in the acid diet. Each diet period lasted 5 days. During each 5-day period, the diets made by different recipes but using the same compositions of natural food materials (listed as an appendix) were served. All food materials were purchased from local supermarkets. Subjects had free access to mineral water.

### Collection of specimens

Twenty-four-hour urine specimens were collected in bottles and stored in a refrigerator during collection. Volume, pH, titration acid, organic acid and creatinine were measured in a sample from urine collected the day before measurement. Four ml urine sample of each experimental day for every person was stored in a deep freezer for later ion analysis.

### Analytical methods

According to Lennon, Lemann Jr. and Litzow (1966)[5], the production of endogenous acid is determined by the sum of 1) the oxidation of sulfate in the sulfur-containing amino acids, 2) the endogenous formation of unmetabolized organic acids, and 3) the net gastrointestinal absorption of alkali produced by the oxidation of organic cations and anions. Using the simplified method proposed by Oh (1989)[6], the net gastrointestinal absorption of alkali (gut alkali) was estimated by the following formula on the basis of urinary ion concentration: [Na^+^+K^+^+Ca^2+^+Mg^2+^]-[Cl^-^+1.8P] (mEq) except for phosphate (mmol). While, the amount of net acid excretion was obtained by measuring (titrable acid + [NH_4_^+^]-[HCO_3_^-^]), because secreted H^+ ^from renal tubules is subjected to two kinds of buffers, phosphate buffer and ammonia buffer. Data necessary for estimation of acid production were collected using HPLC. We used a Hitachi HPLC unit which consists of an intelligent pump L6200, a column oven L5025 and conductivity detector L7470 (Hitachi High-Technologies Corporation. Tokyo, Japan). The separation was performed on a Hitachi GL-IC-C65 column with gourd column GL-IC-C75G for cation and 655-2618 for anion. An aliquot of the filtered and properly diluted urine was injected into an HPLC apparatus with 2.5 mmol H_2_SO_4 _solution as the mobile phase for cation and 2 mmol p-hydroxy benzoic acid/1.5 mmol triethylamine for anion.

Titrable acid was estimated as the amount of 0.1 mol NaOH necessary to titrate back to pH 7.4 from urine pH. Bicarbonate concentration ([HCO_3_^-^]) was calculated using the Henderson-Hasselbach equation for which solubility coefficient of carbon dioxide was taken as 0.0309 mmol/mmHg·L and P_Ka _and P_CO2 _were assumed to be 6.10 and 40 mmHg, respectively. Urine pH was measured at 37°C with pH meter (D-58, Horiba, Kyoto, Japan). Organic acid salts were measured by the Van Slyke and Palmer method with the modification by Lennon, Leman and Litzow (1966)[5]. Briefly, urine was mixed with Ca(OH)_2 _and shaken for precipitating out phosphate. Portions of the filtrate were brought to pH 2.7 at 37°C with 1 mol HCl with a pH meter. The solution then titrated with 0.1 mol NaOH to original pH of the urine to estimate organic acid salts. The organic acid salt measured was corrected for titration of creatinine which was determined with the Folin method. Uric acid was measured by conventional uricase-peroxidase method, using an autoanalyzer.

### Statistical analysis

A series of studies on the same principle has been conducted for three years consecutively. Each year 10, 7 and 9 participants were assigned to either an acid or alkali diet at the beginning of a 5-day study and then switched to the other kind of diet. The first and second diet periods were separated by one month. Because several participants were obliged to discontinue the project due to menstruation, two group populations were treated as independent samples instead of being treated as a paired set of data. Data were presented as mean ± SD. The student t test was used to test the significance of changes in measured parameters between the acid and alkali periods. Differences were assumed to be significant when p < 0.05.

## Results

### Change in urine pH and creatinine excretion

**Figure 1:**
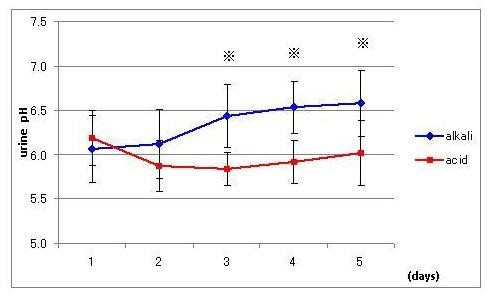
**Effect of acid (square) and alkali (diamond) diets on urine pH. Data are presented as mean ± SD**. Asterisks indicate statistical significance between two groups (p < 0.002).

It took 3 days to reach a steady level of urine pH of 6.5 in the alkali diet and pH 5.9 in the acid diet after switching ordinal daily diets to each designed diet (Figure 1 and Table 1). During all experimental days, the total amount of creatinine excretion remained unchanged around 1000-1100 mg/d, indicating that there is no significant fluctuation of GFR, because creatinine concentration in the serum of healthy young people seems to remain constant.

**Table 1:** Comparison of estimated urinary excretion of ions associated with the acid-base balance which were obtained by averaging data of last 3 experimental days in the acid diet with those in the alkali diet (n = 102).

	alkali diet	acid diet	p
urine volume(L/d)	1.50 ± 0.63	1.37 ± 0.65	NS
pH	6.51 ± 0.34	5.92 ± 0.28	<0.01
ammonium(mmol/d)	24.11 ± 12.78	52.13 ± 13.27	<0.01
phosphate(mmol/d)	24.64 ± 10.37	35.76 ± 14.69	<0.01
sulfate(mmol/d)	9.94 ± 3.25	21.51 ± 6.21	<0.01
uric acid (mg/d)	413.40 ± 81.76	302.84 ± 134.78	<0.01
			

### Relationship between acid generation in the body and acid excretion in urine

**Figure 2:**
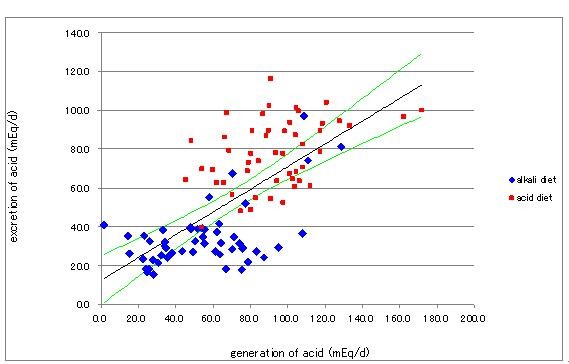
**Relationship between endogenous acid generation and renal acid excretion. Diamonds indicate data for the alkali diet and squares those for the acid diet**. The equation for the straight line adopted to data by the least square method is y = 0.834 × + 27.09 (R^2 ^= 0.362, n = 102, p < 0.01). The lines above and below the regression line are the 99% confidence limits.

In order to confirm the achievement of proper loading of acid, we measured several factors relating with the endogenous fixed acid production and the urinary acid excretion and listed urinary ammonium, phosphate and sulfate together with urinary pH as a typical representative in Table 1. Significant difference in urine [SO_4_^2-^] in the periods of the acid and alkali diets was associated with the amounts of sulfar-containing amino acid in foods of 4262 mg/d in the acid and 2061 mg/d in the alkali diets. Urinary ammonium, phosphate and sulfate were inversely related to the course of urinary pH. On the intake of the alkali diet, these values were significantly lower than those in the acid diet (Table.1). Calculated total effective fixed acid production correlated closely with renal acid excretion, indicating that the metabolic degradation of food materials results in H^+ ^appeared in urine (Figure 2).

### Effect of change in urine pH on the uric acid excretion

**Figure 3:**
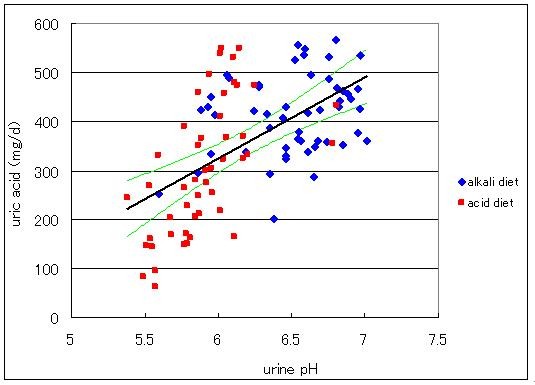
**Relationship between excreted uric acid as expressed in mg uric acid in urine per day and urine pH**. Diamonds indicate data for the alkali diet and squares those for the acid diet. Equation for the straight line obtained by the least square method is y = 165x-669(R^2 ^= 0.342, n = 102, p < 0.01). The lines above and below the regression line are the 99% confidence limits.

Data of excretion of uric acid in urine as expressed as uric acid excretion in mg per day (mg/d) were plotted against urine pH (Figure 3). The amount of excreted uric acid increased with increase in luminal pH. Applying a linear line obtained by the least square method, the amount of uric acid excreted was calculated to be 308 mg/d at pH 5.9 and 407 mg/d at pH 6.5 where specified pH values are corresponding to those steady state values for acid and alkali loading periods. These calculated values are very close to those observed shown in Table 1.

## Discussion

The results obtained in this study are as follows; as urine pH is increased by suppression in the generation of H^+ ^through metabolic degradation of food materials, uric acid excretion increases significantly. The significance of this finding is that although the purine content was less in the alkaline diet compared to the acid diet, the transportable proportion of uric acid (in conjugate base) (P_Ka _= 5.35) was larger in alkaline urine compared with acid urine. These data demonstrate that excretion of uric acid is suppressed in acidic medium. Although Griebsch and Zӧllner (1974)[7] and Clifford, Riumallo, Young and Scrimshaw (1976)[8] have reported that oral purines increase uric acid concentration in serum as well as in urine in dose-dependent manner, we unexpectedly observed the elevation of uric acid excretion in alkali urine while subjects took low-purine diets. This finding strongly suggests that pH-dependent uric acid transport system in human kidney might be intimately committed in excretion of uric acid. Our study provides a key to explain a long standing view that gout is most common among people whose diet is rich in meat and low in vegetable and fruits. Because sulfur-containing amino acids, a main determinant of urine acidity, are contained abundant in animal proteins, people who take diets rich in animal protein will excrete more acidic urine than those having diets low in animal proteins and rich in vegetable and fruits, leading to the difficulty in uric acid removal. Thus, animal protein eaters are more susceptible to gout than vegetable eaters.

Since urine pH could be changed at will by selecting food materials suitable for desired urine pH, one can take preventive procedures for gout without side effects by following appropriate diets.

Because prehistoric Homo sapiens seem to excrete alkaline urine by eating a large amount of high-bicarbonate-yielding plant foods, they would have excreted uric acid more easily than contemporary human being. However, the transition of preagricultural diet to the modern diet with the displacement of plant foods in the pre-historic diet with energy-dense, nutrient-poor foods without buffering capability, such as separated fats, refined sugars and vegetable oil in the contemporary diet, results in a net acid load [9] and makes uric acid excretion difficult. In fact, the Yanomamo Indians eating the vegetable-based diets are reported to have serum uric acid levels in as low as 3 mg/dl even in male which is slightly higher than uric acid observed in primates that express uricase [10]. Although both sulfuric acid and organic acid production rates are lower in the contemporary diet than in the preagricultural diet [9], bicarbonate production is disproportionately lower, thereby shifting urine pH to acid and facing the difficulty of uric acid removal for modern human beings.

## Conclusion

This study has clarified that alkalization of urine by the manipulation of food materials promotes the removal of uric acid. When one pays enough attention to the construction of a nutritionally balanced menu, dietary intervention becomes the safest and the most economical way for the prevention of hyperuricemia.

## Competing interests

The authors declare that they have no competing interests.

## Authors' contributions

AK carried out the analysis of all urine contents and the integration of data into the report. MH participated in the design of the study and helped to draft the manuscript. IS conceived of the study, helped to draft the manuscript and participated in analysis and integration of data.

All authors have read and approved the final manuscript.

## Appendix

The composition of the acid and alkali diets is listed below as consumed amounts, g/day. Energy and protein contents in the acid and alkali diets were 2222 kcal/d and 2212 kcal/d, and 102 g/d and 60 g/d, respectively.

(Alkali diet)

white rice 100 g, rye bread 70 g, pasta 80 g, starch 20 g, hard tofu 100 g, pressed tofu 30 g, fried tofu 6 g, okara 40 g, green soybeans 10 g, milk 150 g, carrot 20 g, leaf vegetable 65 g, tomato 120 g, pepper (red & yellow) 30 g, pumpkin 80 g, green onion 15 g, onion 50 g, cucumber 60 g, cabbage 60 g, lettuce 30 g, garlic 5 g, potato 100 g, aroid 45 g, yam 30 g, mushroom 40 g, banana 45 g, water melon 90 g, walnuts 15 g, dried seaweed 3 g, sugar 4 g, honey 21 g, olive oil 6 g, salad oil 12 g, dressing 10 g, butter 12 g, soy source 9 g, vinegar 3 g, soup prepared from dried bonito and tangle 180 g, alcohol for cooking 15 g, miso (fermented soybeans paste) 9 g, sauce 25 g, chili pepper 1 g, sweet cooking rice wine 4 g, salt 1.3 g, pepper 0.09 g, mayonnaise 12 g.

(Acid diet)

white rice 200 g, bread 90 g, boiled pasta 45 g, starch 6 g, beef round 100 g, cero 90 g, chicken breast fillet 30 g, squid 30 g, egg 100 g, processed cheese 20 g, carrot 50 g, broccoli 20 g, snap pea 20 g, asparagus 20 g, green onion 10 g, bamboo sprout 65 g, corn 25 g, onion 50 g, burdock 15 g, sprout 20 g, tomato source 15 g, soy source 6 g, salt 0.8 g, sweet cooking rice wine 12 g, alcohol for cooking 20 g, pepper 0.09 g, miso 9 g, consommé 1 g, soup prepared from dried bonito and tangle 150 g, mayonnaise 12 g, butter 4 g, salad oil 8 g, sugar 9 g, strawberry jam 20 g.
